# Endocytosis and Endocytic Motifs across the Connexin Gene Family

**DOI:** 10.3390/ijms241612851

**Published:** 2023-08-16

**Authors:** Charles G. Fisher, Matthias M. Falk

**Affiliations:** Department of Biological Sciences, Lehigh University, 111 Research Drive, Bethlehem, PA 18015, USA

**Keywords:** clathrin, connexins, endocytosis, gap junctions, sorting signals

## Abstract

Proteins fated to be internalized by clathrin-mediated endocytosis require an endocytic motif, where AP-2 or another adaptor protein can bind and recruit clathrin. Tyrosine and di-leucine-based sorting signals are such canonical motifs. Connexin 43 (Cx43) has three canonical tyrosine-based endocytic motifs, two of which have been previously shown to recruit clathrin and mediate its endocytosis. In addition, di-leucine-based motifs have been characterized in the Cx32 C-terminal domain and shown to mediate its endocytosis. Here, we examined the amino acid sequences of all 21 human connexins to identify endocytic motifs across the connexin gene family. We find that although there is limited conservation of endocytic motifs between connexins, 14 of the 21 human connexins contain one or more canonical tyrosine or di-leucine-based endocytic motif in their C-terminal or intracellular loop domain. Three connexins contain non-canonical (modified) di-leucine motifs. However, four connexins (Cx25, Cx26, Cx31, and Cx40.1) do not harbor any recognizable endocytic motif. Interestingly, live cell time-lapse imaging of different GFP-tagged connexins that either contain or do not contain recognizable endocytic motifs readily undergo endocytosis, forming clearly identifiable annular gap junctions when expressed in HeLa cells. How connexins without defined endocytic motifs are endocytosed is currently not known. Our results demonstrate that an array of endocytic motifs exists in the connexin gene family. Further analysis will establish whether the sites we identified in this in silico analysis are legitimate endocytic motifs.

## 1. Introduction

Connexins (Cx) are the protein constituents of gap junctions (GJs). All connexins are four-pass transmembrane domain proteins, with their N and C termini located in the cytoplasm. Six connexins oligomerize to form a connexon (a half gap junction channel), which docks head-on with a connexon of an adjacent cell to form a functional gap junction channel [1,2,3]. In general, hundreds to thousands of docked channels cluster together to form a two-dimensional array called a gap junction plaque. Endocytosis of gap junctions typically occur as a whole, with one cell accepting and the other donating the gap junction plaque, resulting in the formation of large cytoplasmic double-membrane vesicles called annular gap junctions (AGJs) or connexosomes (reviewed in [4,5]). Connexins and gap junctions typically have a short half-life of only a few hours [6,7,8]. Hence, efficient mechanisms must exist that regulate and facilitate trafficking of connexins to and removal of gap junctions from the plasma membrane.

Clathrin-mediated endocytosis (CME) is the most prominent endocytic mechanism employed by cells to remove proteins from the plasma membrane (>95%) [9] and has been found to be involved in the endocytosis of Cx43 gap junctions [10,11,12]. Proteins slated for CME require an endocytic motif, which acts as a recruitment site for AP-2 (adaptor protein 2), or another adaptor protein, which, in turn, recruits clathrin. Clathrin interacts with a series of scaffold and membrane bending proteins, which invaginate the plasma membrane inward until the GTPase dynamin can pinch off the growing bud and release it into the cytoplasm as an early endosome [13,14]. In polytopic membrane proteins (to which connexins belong) two types of endocytic motifs have been characterized: (1) tyrosine-based sorting signals of type YXXΦ (where Y is tyrosine, an essential residue that cannot be substituted, even by structurally related phenylalanine or phosphotyrosine residues; X is any amino acid; and Φ is an amino acid with a hydrophobic side chain, such as F, M, L, I, or V) and (2) di-leucine-based sorting signals of type [D/E]XXXL[L/I] (where D and E are aspartic and glutamic acid, respectively; X is any amino acid; and L and I are leucine and isoleucine, respectively), which can bind to specific AP-2 subunits [15,16,17]. In single-spanning membrane proteins, a second type of tyrosine-based sorting signal, NPXY (where N is asparagine, P is proline, X is any amino acid, and Y is tyrosine), has been found [15,17]. Additional experiments in vitro and in vivo have shown that some variability of these canonical signals is tolerated, e.g., the number of unspecified “X” residues between the “Y” and “Φ” residues of the tyrosine-based signal and between the “[D/E]” and “L[L/I]” residues of di-leucine-based signals [18,19,20,21].

Humans contain 21 different connexin isotypes that are specifically expressed in tissue and grouped into five families referred to as alpha, beta, gamma, delta, and epsilon connexins [22,23,24]. Families of connexins are determined by phylogenetic sequence analysis and reflect ancient paralogous gene duplication events [22,25]. Connexin proteins are named according to their molecular weight in kilo Daltons, such as Cx32, Cx43, etc. (capital “X” for humans, lower case “x” for all other species), while the connexin genes are named according to their group, i.e., a1/A1, b1/B1, etc. (see Table 1 for a list of human connexins). Although humans have 21 connexins, that number is typically species-specific. For instance, mice (*Mus musculus*) contain 20 connexins [24], and zebrafish (*Danio rerio*) contain 39 connexins [26]. Varying numbers of connexin isotypes are believed to reflect evolutionary pressures and the need for specialization of these proteins.

Cx43 is the most widely expressed and most well studied connexin. Previously, we found that Cx43 harbors three canonical tyrosine-based endocytic motifs (signals S1 to S3) of the type YXXΦ in its C-terminal cytoplasmic domain (Y^230^VFF, Y^265^AYF, and Y^286^KLV), two of which (S2, Y^265^AYF and S3, Y^286^KLV) have been shown to recruit clathrin and mediate its endocytosis [12,27]. S1 (Y^230^VFF), which is located immediately adjacent to transmembrane domain TM4, was not found to be utilized [27]. This is consistent with the observation that endocytic motifs need to be located at least 6–10 amino acid residues away from a transmembrane domain to allow sufficient space for clathrin-adaptor protein access [15,28]. Ray et al. identified two di-leucine-based endocytic motifs in the C-terminal domain of Cx32—one canonical (E^247^INKLL) and one non-canonical (L^260^KDILR)—and both were found to be important for efficient Cx32 endocytosis [29]. However, similar information is not available for most other connexins. Thus, we examined the amino acid sequences of all 21 human connexins to identify endocytic motifs across the connexin gene family. Interestingly, we found that there is significant variation in endocytic motifs between connexins, including four that do not harbor any recognizable endocytic motif. However, even those connexins are endocytosed into typical annular gap junctions when expressed in HeLa cells.

## 2. Results

### 2.1. Connexin Family Members Contain Both Canonical Tyrosine- and Di-Leucine-Based Endocytic Motifs

To characterize endocytic motifs known to be used in clathrin-mediated endocytosis (tyrosine-based signals (YXXΦ and NPXY) and di-leucine-based signals ([D/E]XXXL[L/I])), all 21 human connexin protein sequences were downloaded from NCBI. Using UniProt, the N-terminal, transmembrane, extracellular and intracellular loops, and the C-terminal domain (CT) were identified for each connexin. Only the I-loop and CT domain were considered for this analysis, as these are the only domains of the connexin proteins that are exposed to the cytoplasm in gap junctions and therefore capable of physically interacting with AP-2 and clathrin.

We found canonical YXXΦ tyrosine-based endocytic motifs in 10 human connexins (five alpha (CX43, CX37, CX50, CX59, and CX62), one beta (Cx30.3), two gamma (CX45 and (CX47), one delta (CX36), and the epsilon connexin (CX23); highlighted in green in Table 1); and canonical [D/E]XXXL[L/I] di-leucine motifs in four human connexins (two beta (CX32 and CX30), one gamma (CX30.2, also known as CX31.3), and one delta (CX31.9); highlighted in yellow in Table 1), but no canonical tyrosine or di-leucine signals were found in the remaining seven human connexins (CX46, CX40, CX26, CX31, CX31.1, CX25, and CX40.1; highlighted in red in Table 1). Additional tyrosine- and di-leucine-based motifs were identified very close to transmembrane-spanning domains in the I loop and the C-terminal domain in several connexins (included in Table 2 in [5]; also see Section 2.6. below). However, based on the observation that endocytic motifs need to be located at least 6–10 amino acid residues away from a transmembrane domain to allow sufficient space for clathrin-adaptor protein access [15,28] and because the S1 tyrosine-based motif located juxtaposed to TM4 is not used in Cx43 endocytosis [27], we did not include them in the present compilation. No NPXY-based signal was found in any human connexin, consistent with the observation that this signal is specific to single-pass transmembrane domain proteins [15].

### 2.2. Non-Canonical Tyrosine and Di-Leucine-Based Endocytic Motifs in Connexins

To explore whether the connexins without canonical tyrosine or di-leucine-based signals may contain a variation of these signals, we performed a second search with less stringent parameters. Kozik et al. found that the number of “X” residues in YXXΦ and di-leucine motifs can vary. The last amino acid in di-leucine motifs can also be valine (V) or arginine (R) [19]. Furthermore, Ray et al. identified a non-canonical endocytic motif with the sequence L^260^KDILR in the C-terminal domain of Cx32 [29] (see Section 3). In accordance with these findings, in our extended analysis we applied the following criteria in searching for additional non-canonical endocytic motifs: (1) tyrosine-based sorting signals were allowed to vary in the number of “X” residues they contained from three to five (YXXXΦ, YXXXXΦ, and YXXXXXΦ), (2) di-leucine motifs were allowed to vary in the number of “X” residues they contained between two to and ([D/E]XXL[L/I], [D/E]XXXL[L/I], [D/E]XXXXL[L/I], and [D/E]XXXXXL[L/I]), (3) the final amino acid of the di-leucine motifs (“L/I”) was also allowed to be “V” or “R”, and (4) we allowed the initial amino acid of the di-leucine motif to be “L” rather than “D/E”. When the search was performed using these less stringent parameters, 14 additional non-canonical signals were identified in 8 connexins (Table 1, motifs in brackets). Importantly, in three of the seven connexins without identifiable motifs, we identified potential non-canonical di-leucine motifs (CX46, DXXLL, and DXXXXXLL; CX40, EXXXLR, and IXXXLL; and CX31.1, LXXXLI, and DXXXXLL). However, no endocytic signals were identified in the C-terminal domain or the intracellular loop domain of CX26, CX31, CX25, and CX40.1, which conformed to the parameters described above.

### 2.3. Location of Endocytic Motifs in Connexins

The majority of the connexins had their endocytic motif located in their C-terminal domain (11 connexins with canonical signals). However, three connexins (CX30.3, CX36, and CX30.2) had canonical signals only located in their intracellular loop domain (Table 1). Two of the connexins with only non-canonical motifs (CX46 and CX31.1) had them located in their C-terminal domain, while we identified non-canonical motifs in both the I loop and the C-terminal domain of CX40 (Table 1).

### 2.4. Conservation of Endocytic Motifs

We reasoned that the endocytic motifs we identified in humans are more likely to represent legitimate signals if they are conserved in other closely related species. By entering the amino acid sequence of all human connexins into NCBI’s BLAST tool, we identified the closest rat homologs, compared them to human connexins, and determined whether the canonical and non-canonical endocytic motifs previously identified in the human connexins also existed in the rat connexins. Of the 33 potential canonical and non-canonical endocytic motifs identified in the twenty-one human connexins, only 6 were not found in both humans and rats. However, this included the only canonical endocytic signals we identified in CX23, CX30.2, and Cx31.9, as well as the two non-canonical di-leucine signals in CX46 (Table 1, underlined motifs). Taken together, these data suggest that most of the endocytic motifs we identified in human connexins are conserved across closely related species (13 of 17 connexins).

### 2.5. Phylogenetic Relation of Endocytic Motifs

**Figure 1:**
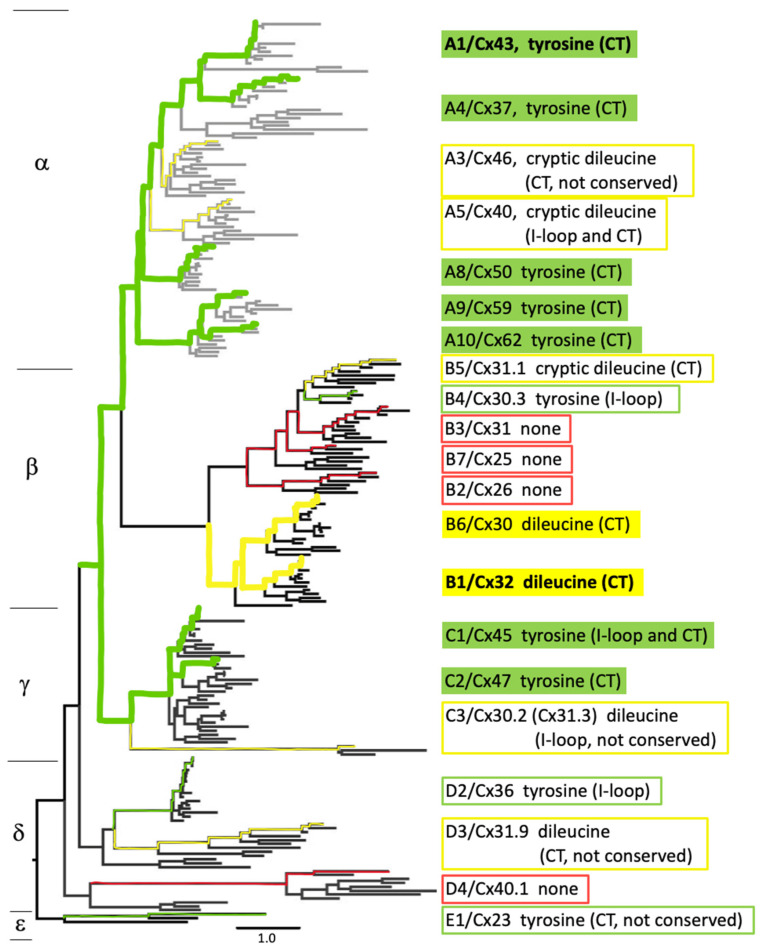
Phylogenetic analysis of endocytic motifs in the connexin gene family. The types of endocytic motifs found in the different connexin family members are highlighted in a phylogenetic tree of the connexin genes [22]. Alpha, beta, gamma, delta, and epsilon groups are marked. Connexins with canonical tyrosine-based endocytic signals located in their C-terminal domain are labeled with solid green. Connexins with canonical di-leucine-based endocytic signals located in their C-terminal domain are labeled with solid yellow. Connexins with tyrosine-based endocytic signals located in the intracellular loop (I loop) or that are not conserved in humans and rodents are indicated by green boxes. Connexins harboring a di-leucine motif in the I loop, that only harbor non-canonical di-leucine motifs (cryptic), or that are not conserved in humans and rodents indicated by yellow boxes. Connexins without identifiable endocytic motifs in their intracellular domains are indicated by red boxes. Endocytic motifs previously shown to be involved in connexin endocytosis (Cx43 and Cx32) are indicated in bold.

To investigate whether the conserved, non-conserved, canonical, and non-canonical tyrosine- and di-leucine-based endocytic motifs we identified across the human connexin family show phylogenetic relationships, we compared the identified signals with an ancestral tree of connexin evolution [22]. Interestingly, well-conserved canonical tyrosine-based endocytic motifs are present in the C termini of closely related alpha connexins Cx43 and Cx37 and closely related alpha connexins Cx50, Cx59, and Cx62 (Figure 1, filled green text boxes). Similarly, conserved, canonical tyrosine-based motifs were found in the C termini of two closely related gamma connexins: Cx45 and Cx47 (Figure 1, filled green text boxes). Moreover, conserved canonical di-leucine-based endocytic motifs were found in the C termini of closely related beta connexins Cx30 and Cx32 (Figure 1, filled yellow text boxes). Three of the four connexins in which we were not able to identify any canonical or non-canonical tyrosine- or di-leucine-based endocytic motifs are the closely related beta connexins Cx31, Cx25, and Cx26 (Figure 1, red outlined text boxes). All other endocytic signals we identified that were not located in the C-terminal domain but instead in the I loop were not conserved or were non-canonical (cryptic) motifs (Figure 1, green and yellow outlined text boxes). Thus, C-terminal-located, well-conserved tyrosine-based endocytic signals appear to be preferentially present in the alpha and gamma subfamilies, while two of the beta connexins have well-conserved C-terminal di-leucine signals. The endocytic signals in the other beta connexins and the gamma, delta, and atypical epsilon connexins appear much less well defined. Based on these phylogenetic relationships, one can speculate that the identified non-canonical di-leucine motifs in the alpha connexins, Cx46 (not conserved) and Cx40, may not be legitimate endocytic signals that are utilized. Similarly, the non-canonical tyrosine-based signal identified in the I loop of the beta connexin, Cx30.3; the non-canonical, non-conserved di-leucine motif identified in the I loop of the gamma connexin, Cx30.2/(Cx31.3); and the non-canonical tyrosine- and di-leucine-based endocytic signals we identified in the delta connexins, Cx36 and Cx31.9, and the epsilon connexin, Cx23, may not be used as well.

### 2.6. Conserved Canonical and Non-Canonical Di-Leucine-Type Motifs Located in the TM4/CT Transition Zone

**Figure 2:**
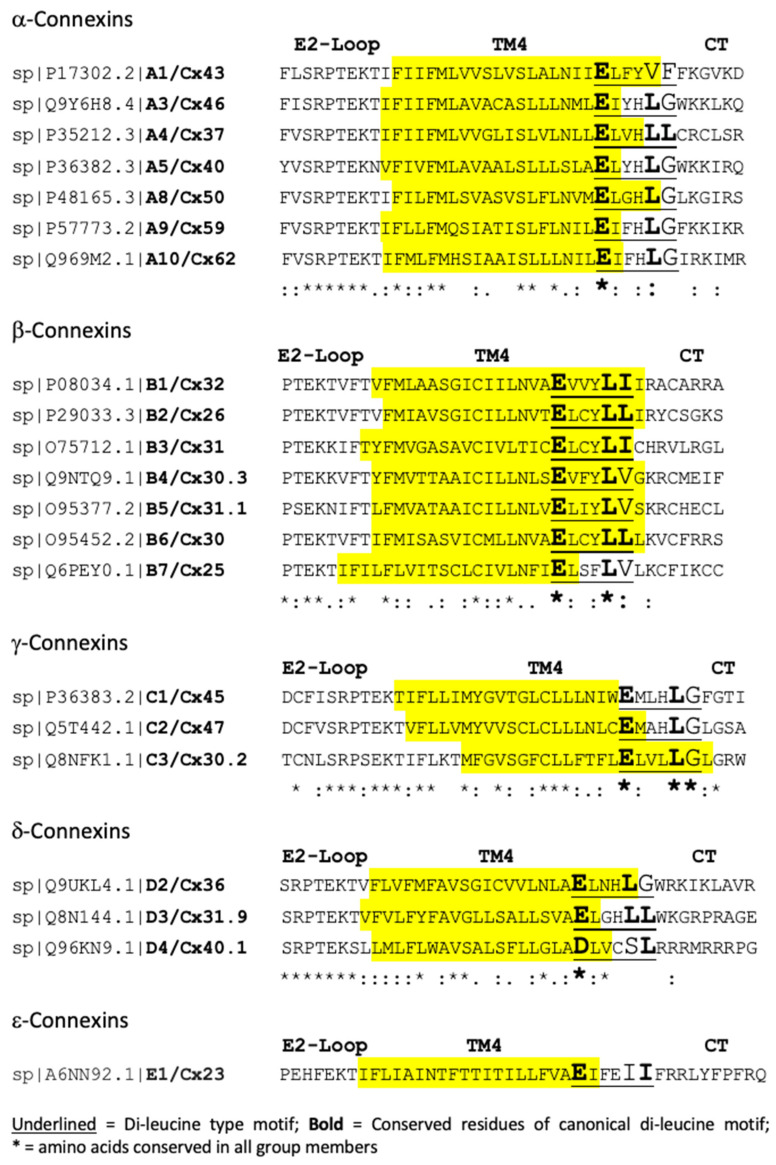
A motif of an unknown function resembling a di-leucine-based endocytic motif is present at the end of TM4 in all human connexins. Connexins are grouped according to their clade. Transmembrane domain 4 (TM4, highlighted in yellow) and portions of the flanking second extracellular loop (E2-loop) and C-terminal domain (CT) are shown. Di-leucine-type motifs are underlined, and conserved residues of the canonical di-leucin motif (D or E, and L and I) are indicated in bold. Amino acids conserved in all group members are indicated with an asterisk. Note the presence of “LG” residues in alpha and gamma connexins and “LL, LI, and LV” residues in beta connexins.

When compiling the endocytic motifs, we excluded motifs that were located within 10 amino acids of transmembrane domains, as it was reasoned that these amino acids would not be accessible to the endocytic machinery [15]. However, all human connexins have a canonical or non-canonical (cryptic) di-leucine-type motif in the TM4/C-terminal domain transition zone (Figure 2). Six connexins (Cx37, Cx32, Cx26, Cx31, Cx30, and Cx31.9) have canonical di-leucine motifs, while the others have atypical amino acids in the last position (Cx46, Cx40, Cx50, Cx59, Cx62, Cx30.3, Cx31.1, Cx25, Cx45, Cx47, Cx30.2, and Cx36), second last position (Cx40.1 and Cx23), or in both positions (Cx43) (Figure 2). Interestingly, “LG”, as the last two residues, is almost exclusively conserved in the alpha and gamma connexins (except for Cx43 and Cx37), while canonical residues “L[L/I]” are present in most beta connexins (Cx32, Cx26, Cx31, and Cx30), and “LV” residues are present in the remaining beta connexins (Cx30.3, Cx31.1, and Cx25). In all connexins, the first amino acid of the motif is glutamic acid (E), and only Cx40.1 has an aspartic acid (D) residue. In all these motifs, the central region consists of three undefined “X” residues, as is typical for [D/E]XXXL[L/I] di-leucine-type motifs. While these motifs are likely not involved in connexin endocytosis due to their proximal membrane location, which interferes with clathrin-adaptor accessibility [15], their role and reason for conservation are currently not known. However, when Ray et al. mutated this domain in Cx32, surprisingly, its endocytosis was increased [29] (see Section 3).

### 2.7. Connexins with and without Identifiable Endocytic Motifs Endocytose to Form Typical Cytoplasmic Annular Gap Junctions

To test whether connexins with and without identifiable endocytic motifs can be endocytosed and form annular gap junctions, we transfected HeLa cells with different connexin types tagged with GFP on their C terminus. We chose HeLa cells, as they do not express endogenous gap junctions [30], to eliminate the possibility that an endogenously expressed connexin would interact with clathrin and “passively” internalize the GFP-tagged connexin.

**Figure 3:**
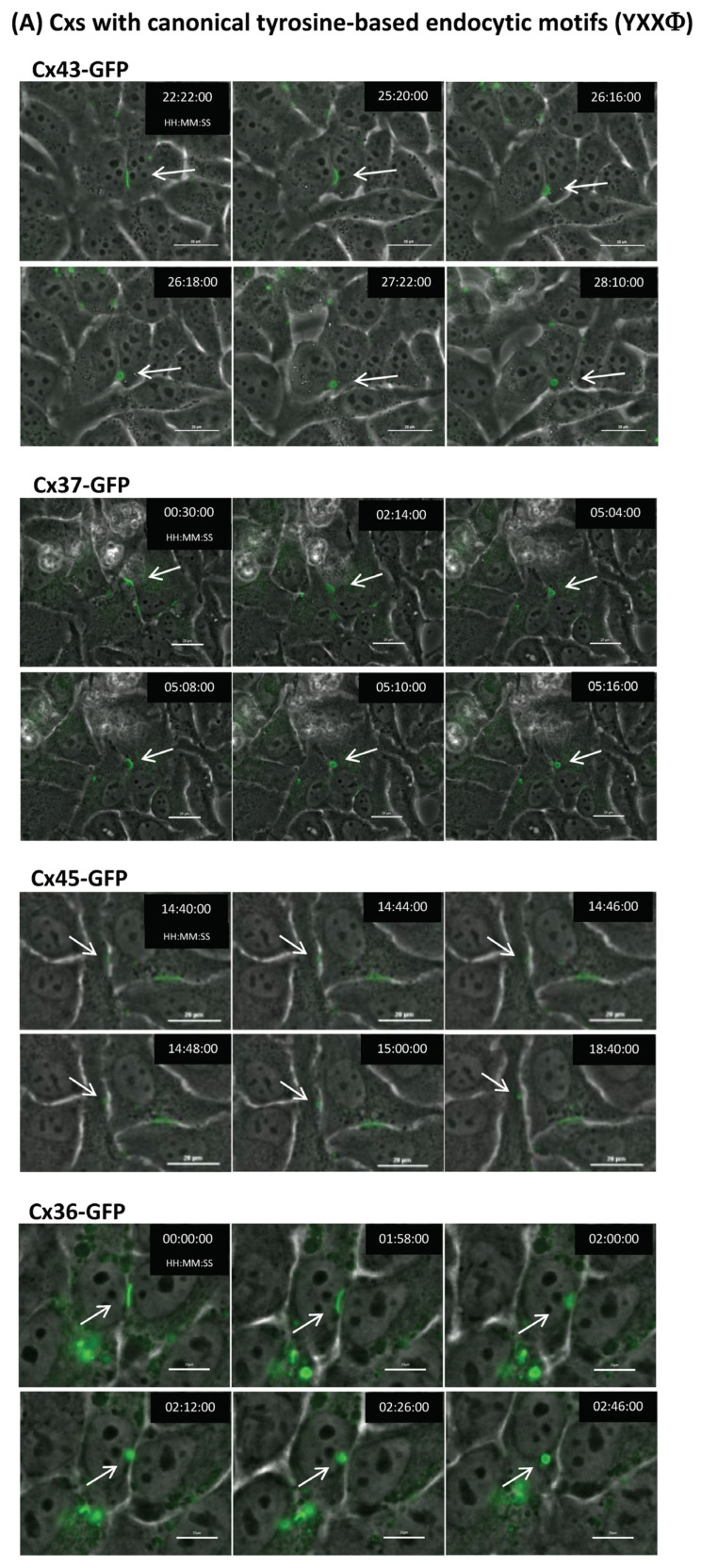

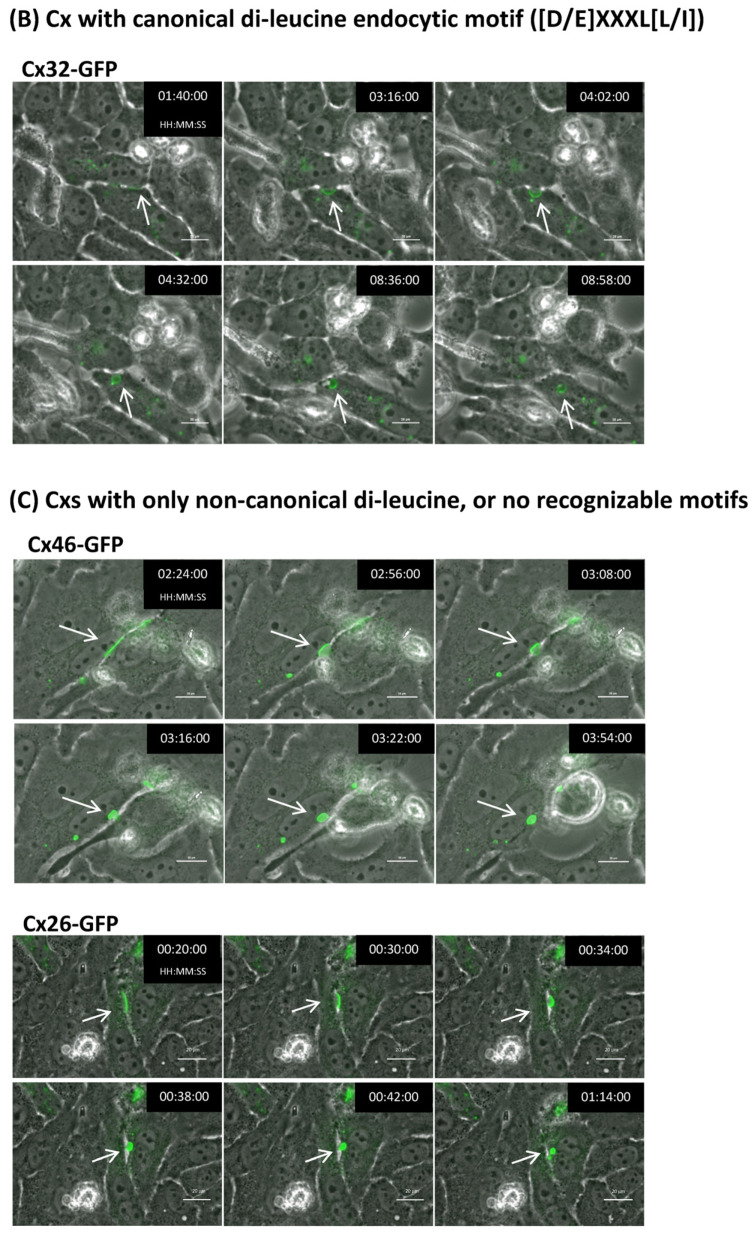
Connexins with and without recognizable endocytic motifs can be endocytosed in HeLa cells. Connexins tagged with GFP on their C terminus were transfected into connexin-deficient HeLa cells and followed for 24–48 h using time-lapse microscopy. Images acquired at selected time points are shown. All connexins formed gap junction plaques in the plasma membranes, and all gap junctions, irrespective of connexin type (with or without identifiable endocytic motifs), endocytosed into typical annular gap junctions (connexosomes) with similar appearance and dynamics (marked with arrows). Time stamp is in hours: minutes: seconds. Scale bars = 20 μm.

We expressed representative connexins from each group, including Cx43 and Cx37 (both tyrosine-based endocytic motifs located in the C-terminal domain), Cx36 (tyrosine-based signal located in the I loop), Cx45 (tyrosine-based motifs in both the I loop and the C-terminal domain), Cx32 (di-leucine-based motif located in the C-terminal domain), Cx46 (only non-canonical di-leucine motifs in the C-terminal domain), and Cx26 (no identifiable endocytic motifs) (Table 1). Then, 24 h post transfection, cells were placed into a Nikon BioStation live cell imager, and gap junction plaques were selected and followed for 48 h or until the plaques had been internalized. We found that all connexins, including Cx46 and Cx26, endocytosed into typical annular gap junctions with similar overall kinetics (Figure 3). Once the selected plaques visibly started to bulge inward, scission and annular gap junction vesicle formation were completed in all connexins within approximately 30 min (Figure 3). Whether or not some of the connexin types may have exhibited delayed endocytosis could not be evaluated in this analysis, as it is not known how long the gap junction plaques selected for imaging were present in the membrane before we began to follow them using time-lapse imaging. Taken together, these data indicate that connexins, regardless of whether they contain identifiable endocytic motifs (canonical or non-canonical), can endocytose into annular gap junctions.

## 3. Discussion

Here, we analyzed the expression of well-known sequence motifs that serve as binding sites for clathrin adaptors such as AP-2 (termed tyrosine- and di-leucine-based sorting signals) in all 21 human connexins. We found that canonical tyrosine-based sorting signals (YXXΦ) are present in the cytoplasmic domains (intracellular loop and C-terminal domain) in 10 connexins, 4 additional connexins harbor canonical di-leucine-based motifs ([D/E]XXXL[L/I]), and the remaining 7 connexins only harbor non-canonical di-leucine motifs (shorter or longer “X” residue core or atypical amino acid residues in position 1 or 6) (3 connexins) or do not harbor any identifiable endocytic motif in their cytoplasmic domains at all (4 connexins) (Table 1, green, yellow, and red grouping). Most connexins have their endocytic motif located in the C-terminal domain (12 connexins), while three have endocytic motifs in their I-loop domain (Cx30.3, Cx36, and Cx30.2), and two have endocytic motifs in both domains (Cx45 and Cx40) (Table 1). Only in four connexins (Cx23, Cx30.2, Cx31.9, and Cx46) were the motifs not conserved between humans and a closely related species (rat) (Table 1), suggesting that most of the identified signals are legitimate endocytic signals that can be used in connexin endocytosis. Phylogenetic analyses indicated that tyrosine-based motifs are present in closely related connexins, preferentially in the alpha and gamma group, while di-leucine motifs are present preferentially in closely related beta connexins (Figure 1). The remaining beta, gamma, delta, and epsilon connexins are more variable and only express non-canonical motifs or endocytic motif types that have not yet been identified (Figure 1). Interestingly, representative connexins from different groups all endocytosed into typical annular gap junctions when expressed in HeLa cells (Figure 3). This was also the case for connexins that lacked any canonical or non-canonical endocytic motifs, which hints at the possible use of alternative clathrin adaptors (adaptors that recruit clathrin independent of tyrosine- or di-leucine-based sorting signals) or, in some cases, the possible use of alternative, non-clathrin-based endocytic pathways (e.g., connexins without identifiable endocytic motifs).

Detailed information on the endocytosis of some connexins exists. For example, Cx43 has been found to interact with and use clathrin for Cx43 gap junction internalization [10,12]. Cx43 harbors three canonical tyrosine-based endocytic motifs (YXXΦ and signals S1 to S3) in its C-terminal domain (Y^230^VFF, Y^265^AYF, and Y^286^KLV), of which two (S2, Y^265^AYF and S3, Y^286^KLV) can recruit clathrin and mediate Cx43 gap junction endocytosis [27,31]. Due to its juxtaposed membrane location and consistent with the observation that endocytic YXXΦ motifs need to be located at least 6–10 amino acid residues away from a transmembrane domain [15,28], signal S1 (Y^230^VFF) was not found to be utilized [27]. Cx30 and Cx31.1 were also shown to interact with clathrin, which was required for their degradation [32,33], and Cx26 has been shown to require dynamin (a GTPase involved in pinching off the endocytic invagination from the plasma membrane) for degradation [34]. However, the use of dynamin is not restricted to clathrin-mediated endocytosis and is also used in other, non-clathrin-mediated endocytic pathways. Cx32 has been shown to colocalize with clathrin and AP-2 in retrovirally infected Cx32-expressing cells (BxPC3 and LNCaP: pancreatic cancer and prostate cancer cell lines, respectively) [29]. Di-leucine-based motifs were identified in Cx32 at E^247^INKLL (canonical) and L^260^KDILR (non-canonical). When mutated, both motifs resulted in the formation of larger gap junction plaques. Intriguingly, while E^247^INKLL conforms to the classical definition of a di-leucine-based motif ([D/E]XXXL[L/I]), L^260^KDILR does not, indicating that Cx32 may employ non-canonical (cryptic) endocytic motifs for its endocytosis. We identified three other connexins that only harbor non-canonical di-leucine-based endocytic motifs (Cx46, Cx40, Cx31.1), suggesting that these connexins may use them for endocytosis as well.

Rat Cx46 has been shown to undergo endocytosis in HeLa cells, and through sequential deletion mutagenesis of the C terminus, an approximately 25 amino acid region (P284-A311) has been identified that is required for efficient endocytosis, despite this region lacking any identifiable endocytic motifs [35]. We identified four connexins that appear not to harbor any identifiable endocytic motif in their intracellular domains located sufficiently far away from transmembrane domains to allow for clathrin-adaptor interaction [15] (Cx26, Cx31, Cx25, and Cx40.1), suggesting that these connexins, including Cx46, may use alternative clathrin adaptors or even non-clathrin-based endocytic pathways.

Cx43, in addition to utilizing tyrosine-based sorting signals, has been suggested to utilize ubiquitin as an alternative means of endocytosis [36,37,38,39]. Cx43 in gap junction plaques can be polyubiquitinated in a K63-linked pattern [40], which can serve as an internalization signal [41,42]. Ubiquitination is carried out by E3 ubiquitin ligases [41], and Cx43 has been shown to interact with the E3 ubiquitin ligases Nedd4-1, Wwp1, Smurf2, and Trim21 [43,44,45,46]. Nedd4, in particular, is known to add K63-polyubiquitin chains to target proteins [47], and its interaction with Cx43 has been shown to be regulated via Cx43 serine279/282 phosphorylation [48]. Cx36 has also been shown to interact with ubiquitin ligases [49], and Cx45 has been reported to interact with Nedd4 [50]. Eps15, an alternative clathrin adaptor protein, can recognize and bind to K63 ubiquitin chains and recruit clathrin independent of tyrosine and di-leucine-based sorting signals [46], and it has been shown to interact with and bind to Cx43 [37]. Eps15, as well as other alternative clathrin-adaptor proteins (CLASPs), is a component of clathrin-coated pits and vesicles [51,52], and Eps15 has been reported to interact directly with AP-2 [53]. In addition, Eps15 can couple ubiquitinated cargo to clathrin-independent internalization [54]. Furthermore, Cx36 has been shown to interact with caveolin-1 in a calcium-regulated manner, and it has been suggested to play a role in the fast, steady-state endocytosis of Cx36 [55]. Hence, it is possible that connexins 46, 26, 31, and 40.1 (and potentially other connexins) use Eps15 (or another adaptor) and K63-based polyubiquitination or even a caveolin-dependent pathway (or another endocytic pathway) for endocytosis.

Ray et al., in addition to identifying canonical and non-canonical di-leucine motifs in the C-terminal domain of Cx32 (described above), identified a di-leucine-type motif, E^209^VVYLI, in Cx32, which is located at the junction between transmembrane domain 4 and the C-terminal domain [29]. Due to its juxtaposed membrane location, it is unlikely to serve as a clathrin-adaptor binding site [15]. Consistent with this, when the sequence was mutated, it was found to enhance (not to reduce) the endocytosis of Cx32 by an undefined mechanism. Indeed, all connexins harbor a canonical or non-canonical di-leucine-type motif between TM4 and the C-terminal domain (Figure 2). While its purpose is currently unclear, its strict conservation in all connexins suggests an important role in gap junction function.

Taken together, all connexins we expressed in HeLa cells endocytosed into typical annular gap junctions with remarkably similar appearance and kinetics (Figure 3), a finding that suggests that a consistent endocytic pathway is utilized for endocytosis across the connexin protein family, although some of these connexins are lacking recognizable endocytic motifs. Our work of analyzing endocytic motifs across the connexin gene family contributes to a better understanding of gap junction endocytosis. Further analysis will establish whether the sites we identified are indeed legitimate endocytic motifs.

## 4. Materials and Methods

### 4.1. Endocytic Motif Search

Human connexin amino acid sequences were obtained and downloaded from the National Center for Biotechnology Information (NCBI) database at the National Library of Medicine (https://www.ncbi.nlm.nih.gov (accessed on 1 June 2023)). The UniProt database (EMBL-EBI, https://www.uniprot.org (accessed on 1 June 2023)) was used to determine the N-terminal domain, the four transmembrane-spanning domains (TM1-4), the extracellular loop domains (E1 and E2 loop), the intracellular loop domain (I loop), and the C-terminal domain (CT) in each connexin. To search for canonical and non-canonical endocytic motifs, individual types of amino acids (Y, D, E, etc.) were highlighted in Microsoft Word; then, potential endocytic signals were assessed manually, making sure they conformed with specified sequence criteria and that they were located in the I-loop or C-terminal domain. Sequence alignments were carried out using CLUSTAL OMEGA (http://www.clustal.org/omega/ (accessed on 1 June 2023)) and the Basic Local Alignment Search Tool (BLAST) (https://blast.ncbi.nlm.nih.gov/Blast.cgi (accessed on 1 June 2023)) platforms.

### 4.2. Live Cell Imaging

Connexin-deficient HeLa cells (cat. no. CCL-2; American Type Culture Collection [ATCC], Manassas, VA, USA) were maintained at 37 °C under 5% CO_2_ in low-glucose DMEM. Media were supplemented to a final concentration of 10% with fetal bovine serum (FBS), 2 mM L-glutamine, 50 IU/mL penicillin, and 50 μg/mL streptomycin. For transfection experiments, HeLa cells were plated onto 35 mm diameter fibronectin-coated glass-bottom dishes (0.170 mm thickness: Mattek, Ashland, MA, USA) to reach 75–80% confluency within 24 h. Cells were transfected with Lipofectamine2000 (cat. no. 11668027; Invitrogen, Carlsbad, CA, USA) as recommended by the manufacturer. Cells were placed in a Nikon BioStation IM-Q system with a 40× air objective (Nikon Instruments Inc., Melville, NY, USA). Fluorescence and phase contrast images were acquired every 2 min for 24–72 h. Time-lapse image sequences were annotated using NIS Elements AR 3.00 (Nikon Instruments Inc., Melville, NY, USA).

## Figures and Tables

**Table 1:** Tyrosine and di-leucine-based endocytic signals in human connexins. Domains of all 21 human connexin proteins exposed to the cytoplasm (intracellular loop and C-terminal domain) were searched for tyrosine-based (YXXΦ) and di-leucine-based ([D/E]XXXL[L/I]) endocytic motifs. In addition, known non-canonical motifs (variation in the length of the “X” core and the first and last amino acid residues in di-leucine motifs) (in brackets) were characterized. Connexins with tyrosine-based endocytic motifs are highlighted in green, connexins with di-leucine-based motifs are highlighted in yellow, and connexins harboring only non-canonical endocytic motifs or not harboring any recognizable endocytic motif are highlighted red. Connexins that expressed in this work are labeled with *. Endocytic motifs previously shown to be involved in connexin endocytosis (Cx43 and Cx32) are indicated bold. Non-canonical motifs are in parentheses. Motifs not conserved in humans and rodents are underlined.

Cx Gene Name	Cx Protein Name	Type of Endocytic Signal	Location of Endocytic Signal	
			Intracellular-Loop	C-Terminal Domain
A1	CX43 *	Tyrosine based (YXXF)	---	Y^230^VFF; **Y^265^AYF**; **Y^286^KLV**
A4	CX37 *		---	Y^266^LPV; Y^281^NGL
A8	CX50		---	Y^266^QLL; Y^278^FPL; (Y^316^QETL); Y^323^AQV
A9	CX59		---	(D^283^YNLL); Y^284^NLL; Y^297^PSL; (L^500^TNNLI)
A10	CX62		---	(L^433^SRLL); Y^513^VCV
B4	CX30.3		Y^117^DNL	---
C1	CX45 *		Y^141^PEM	Y^301^TEL
C2	CX47		---	Y^330^SLV
D2	CX36 *		Y^109^STV	---
E1	CX23		---	Y^200^FPF
B1	CX32 *	Di-leucine based	(E^103^KKMLR)	**E^247^INKLL; (L^260^KDILR)**
B6	CX30		---	E^243^MNELI
C3	CX30.2 (CX31.3)		E^111^EETLI	---
D3	CX31.9		---	E^233^AQKLL
A3	CX46 *	Non-canonical or Not identifiable	---	(D^296^FKLL); (E^367^AGAAPLL)
A5	CX40		(E^105^KRKLR)	(L^145^QGTLL)
B2	CX26 *		---	---
B3	CX31		---	---
B5	CX31.1		---	(L^245^SGDLI); (D^255^SHPPLL)
B7	CX25		---	---
D4	CX40.1		---	---

* = Expressed here; Bold = Shown previously to use this motif; (…) = Non-canonical motif; Underlined = Not conserved between human and rodents.
